# The duration of disgusted and fearful faces is judged longer and shorter than that of neutral faces: the attention-related time distortions as revealed by behavioral and electrophysiological measurements

**DOI:** 10.3389/fnbeh.2014.00293

**Published:** 2014-08-27

**Authors:** Dandan Zhang, Yunzhe Liu, Xiaochun Wang, Yuming Chen, Yuejia Luo

**Affiliations:** ^1^Institute of Affective and Social Neuroscience, Shenzhen UniversityShenzhen, China; ^2^State Key Laboratory of Cognitive Neuroscience and Learning, Beijing Normal UniversityBeijing, China; ^3^School of Kinesiology, Shanghai University of SportShanghai, China

**Keywords:** threat-related, time perception, disgust, fear, event-related potential

## Abstract

Time perception has been shown to be altered by emotions. This study employed event-related potentials (ERPs) to examine the effects of two threat-related emotions on the judgment of time intervals in the range of 490–910 ms. We demonstrated that disgust and fear have distinct influences on time perception. At the behavioral level, disgusted faces were estimated longer and fearful faces were estimated shorter (i.e., the generalization gradient for the disgusted faces was shifted left while the generalization gradient for the fearful faces was shifted right) when compared with neutral faces. Accordingly, the contingent negative variation, an online ERP index of timing, displayed larger area in disgust and smaller area in fear conditions when compared with neutral condition (disgust = 1.94 ± 2.35 μV_•_s, neutral = 1.40 ± 2.5 μV_•_s, and fear = 1.00 ± 2.26 μV_•_s). These findings indicated that specific neural mechanisms may underlie the attention effects of different subtypes of threat-related emotions on timing; compared with neutral faces, fearful faces are likely to attract more attentional resources while disgusted faces may attract less attentional resources for emotional processing. The major contribution of the current study is to provide neural correlates of fear *vs*. disgust divergence in the aspect of time perception and to demonstrate beyond the behavioral level that the categorization of threat-related emotions should be refined so to highlight the adaptability of the human defense system.

## Introduction

The perception of time is essential for the survival of individuals and for everyday activities (Wittmann, 2009; Mella et al., 2011). Humans possess neural mechanisms which enable them to estimate time accurately. However, perceptual time is usually not isomorphic to physical time and time perception has been shown to be altered by emotion (Droit-Volet and Meck, 2007; Droit-Volet and Gil, 2009). In particular, our representations of time interval are easily distorted by internal emotional states or external emotional events/stimuli, which may change the perceived length of time and make it flies past quickly or drags slowly (Gil and Droit-Volet, 2011). Although time perception in various emotional conditions is part of everyday experience, analysis of the intricate interplay between emotion and interval timing remains relatively rare (Schirmer, 2004; Droit-Volet and Meck, 2007; Wittmann, 2009). Studying the temporal illusions caused by emotion is a means of gaining a better understanding of the function of emotions and the mechanism underlying their influence on behaviors (Droit-Volet and Gil, 2009).

The psychological mechanism of time perception is usually explained using the pacemaker-accumulator model (Gibbon et al., 1984). In brief, the model includes an internal pacemaker that sends pulses to an accumulator. The longer the stimulus duration, the more pulses are accumulated, and the longer the subjective duration is judged to be. Attending to non-temporal information (e.g., a concurrent task) is thought to divert processing resources from the timer and to reduce the number of pulses sent to the accumulator, thus resulting in an underestimation of time (Angrilli et al., 1997; Brown, 1997; Fortin, 2003; Coull et al., 2004; Droit-Volet et al., 2004; Buhusi and Meck, 2006; Meck and MacDonald, 2007). On the contrary, the duration of a given stimulus may be judged longer than its physical duration if additional attentional resources are allocated to timing. The current study was designed to examine the attention-related time distortions caused by emotions. Considering that emotion may also influence time perception through the arousal mechanism (Droit-Volet and Meck, 2007; Stetson et al., 2007; Grommet et al., 2011; Lambrechts et al., 2011), the arousal of the emotional stimuli in this study was carefully controlled so as to prevent our results from being contaminated by the arousal differences among emotional conditions.

Moreover, many studies have suggested that the distortion of emotions on time perception is usually stronger for negative than for positive emotional stimuli (Angrilli et al., 1997; Noulhiane et al., 2007), which might be explained by the higher biological relevance of unpleasant stimuli associated with threatening situations (Droit-Volet and Meck, 2007; Pessoa, 2008; Droit-Volet and Gil, 2009; Droit-Volet et al., 2010). Threat-related emotions are typically associated with enhanced attention (Vuilleumier et al., 2001; Pessoa et al., 2002; Vuilleumier, 2005; Williams, 2006). However, the model of threat-related processing is usually oversimplified with almost exclusive focus on the emotion of fear (Woody and Teachman, 2000; Vaish et al., 2008). It is more and more recognized that threat-related emotions should be examined separately because each discrete emotion may have a specific function and consequently a specific attention-related effect on time perception (Frijda, 1988; Vaish et al., 2008; Droit-Volet and Gil, 2009; Krusemark and Li, 2011). However, direct comparison of time perception between different threat-related emotions remains very few (see Gil and Droit-Volet, 2012 and Tipples, 2008 for the limited examples). As the most frequently studied threat-related emotion, fear denotes dread of impending danger and an intense urge to defend oneself, primarily by getting out of the situation (Vaish et al., 2008). As another threat-related emotion, disgust represents certain set of stimuli that signify potential danger in our environment, e.g., rotting food or dirty animals, which would contaminate individuals both physically and psychologically (Oaten et al., 2009). Previous researches have demonstrated that fear and disgust could induce divergent physiological responses and cognitive processes: disgust tends to activate parasympathetic system and suppresses action while fear stimulates sympathetic pathways and prompts fight or flight (Ekman et al., 1983; Levenson, 1992); disgust provokes instant sensory rejection so as to prevent people from biological/psychological contamination as soon as possible, whereas fear quickly attracts attention so as to ensure sensory acquisition (Jones, 2007; Susskind et al., 2008). Since both fear and disgust tend to promote avoidance behaviors (Hutcherson and Gross, 2011), the present study investigated and compared the time perception of these two subtypes of threat-related emotions.

Finally, it is believed that the neural procedure and characteristics of the time perception of different threat-related emotions could be clearly disclosed using the event-related potential (ERP) technique, which has a high time resolution and could follow the neural dynamics timely. To date, however, there are very few studies investigating the ERP patterns of the effect of emotion on time perception, except one very preliminary study of our group (Gan et al., 2009).

The aim of the present study was to investigate the ERPs recorded during the temporal generalization task and to examine the effects of two threat-related emotions on the judgment of time duration using arousal-controlled emotional stimuli. Considering that while fearful stimuli attract attention, disgusting stimuli are assumed to suppress attention (Vuilleumier, 2005; Krusemark and Li, 2011), it was expected that fear and disgust may have distinct influences on attention-related mechanism of time perception and thus may result in different ERP patterns when participants watch and estimate the time duration represented by fearful and disgusting stimuli. Since facial expressions play an essential role in social communication, this study employed emotional faces as the targets of time perception. Accordingly, we investigated the face-sensitive N170 component, as well as its positive counterpart component of vertex positive potential (VPP) in the ERP data. Both ERP components have been proved to be modulated by emotional faces, with larger amplitudes elicited in response to emotional, compared with neutral, facial expressions (Batty and Taylor, 2003; Blau et al., 2007; Schyns et al., 2007; Zhang et al., 2013). More importantly, a slow negative potential, namely the contingent negative variation (CNV), has been shown in numerous studies of time perception to be an online index of subjective timing (Walter et al., 1964; Macar and Vidal, 2004; but see Kononowicz and van Rijn, 2014). The CNV amplitude correlates positively with the length of the estimated duration (Macar and Vidal, 2003). It is expected that early ERP components such as N170 and VPP would be enhanced by emotions, and that the amplitudes of the slow CNV component may display separated waveforms in fear *vs*. disgust conditions, indicating specific neural mechanisms underlying the attention effects of different subtypes of threat-related emotions on time perception.

## Methods

### Participants

Twenty-nine healthy subjects (15 females; age range = 21 to 26 years) were recruited from Beijing Normal University in China as paid participants. All subjects were right-handed and had normal or corrected-to-normal vision. They gave their written informed consent prior to the experiment. The experimental protocol was approved by the local ethics committee (Beijing Normal University).

### Stimuli

The stimuli used for the representation of duration were a gray oval and three types of pictures, namely, disgusted, fearful, and neutral faces. Facial pictures were black and white photographs selected from the native Chinese Facial Affective Picture System (CFAPS) (Gong et al., 2011), with equal number of facial pictures between males and females. A total of 60 faces were used (20 disgusted, 20 fearful, and 20 neutral faces). Each picture had been assessed for its valence and arousal on a 9-point scale with a large sample of Chinese participants in a previous survey. The ANOVA performed on the average scores showed that the two categories of negative faces significantly differed from neutral faces in valence [*F*_(2, 57)_ = 180, *p* < 0.001, η^2^_p_ = 0.863; mean ± standard deviation: disgust = 3.24 ± 0.33, fear = 3.06 ± 0.38, neutral = 4.81 ± 0.24; disgust/fear *vs*. neutral: *p*s < 0.001] while the arousal ratings of the three categories of faces did not show any significant difference [*F*_(2, 57)_ = 2.05, *p* = 0.138, η^2^_p_ = 0.067; disgust = 5.69 ± 0.45, fear = 5.78 ± 0.52, neutral = 5.50 ± 0.35; disgust *vs*. neutral: *p* = 0.550; fear *vs*. neutral: *p* = 0.156]. Of note, to prevent our results from being contaminated by the arousal across three emotional conditions, the 20 neutral faces were selected to have a relatively higher arousal score compared with most of the neutral faces in the CFAPS [there were a total of 422 neutral faces in the CFAPS (valence = 4.29 ± 0.52; arousal = 3.84 ± 0.69)].

### Procedure

Participants were seated in a dimly lit and sound-attenuated room. Stimuli were presented on a LCD monitor at a viewing distance of approximately 100 cm. Stimulus display and behavioral data acquisition were conducted using E-Prime 2.0 (Psychology Software Tools, Inc., Pittsburgh, PA). All stimuli were presented with the same contrast and brightness on the black background (4.0 × 4.6° visual angle).

**Figure 1:**
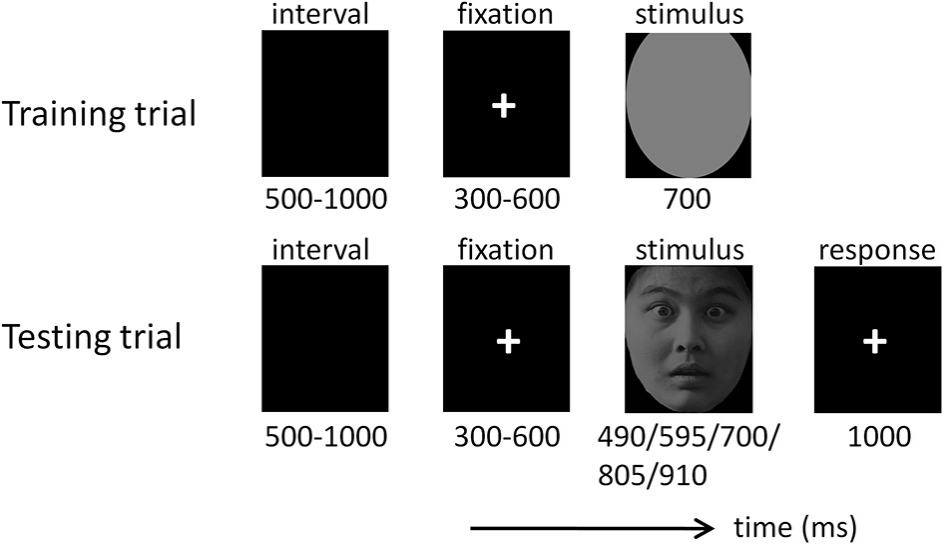
**Illustration of one training trial and one testing trial in this study**.

The temporal generalization procedure is one of the most frequently employed tasks when investigating time perception (Pouthas et al., 2000; Macar and Vidal, 2003, 2004; Gil and Droit-Volet, 2011). It involves comparing a presented duration (equal or not equal to) with a standard duration stored in memory. The temporal generalization task was composed of two phases—a training phase and a testing phase (Figure 1). In the training phase, participants watched the “standard” stimulus duration (700 ms) 10 times, represented by a gray oval, which had the same size as the emotional faces. In the testing phase, participants watched a series of comparison durations (490, 595, 700, 805, and 910 ms; see also Pouthas et al., 2000; Pfeuty et al., 2003b) presented in the form of disgusted, fearful, or neutral faces. Participants were required to judge whether the presented duration of the face was “the same” or “not the same” as the standard duration by pressing the “F” or “J” button (yes or no) on the computer keyboard with their left or right index finger. The assignment of keys to “yes” and “no” responses was counterbalanced across participants.

The testing phase consisted of 15 blocks (each containing 40 testing trials). Each face was presented twice in each of the five duration conditions in a random order. The emotion (disgust, fear, and neutral) of faces varied randomly across trials. The standard stimulus was presented five times at the beginning of each block to prevent the participants from forgetting it. Blocks were separated by self-terminated breaks. Responses with latencies less than 1000 ms were considered valid.

### EEG recording and analysis

Brain electrical activity was recorded referentially against left mastoid and off-line re-referenced to the average of the left and right mastoids, by a 64-channel amplifier with a sampling frequency of 250 Hz (NeuroScan Inc., Herndon, USA). Besides electrooculogram electrodes, a 62-channel electroencephalography (EEG) data were collected with electrode impedances kept below 5 kΩ. Ocular artifacts were removed from EEGs using a regression procedure implemented in NeuroScan software (Scan 4.3).

The data analysis and result display in this study were performed using Matlab R2014a (MathWorks, Natick, USA). The recorded EEG data were filtered with a 0.01–30 Hz finite impulse response filter with zero phase distortion. Filtered data were segmented beginning 200 ms prior to the onset of stimulus and lasting for 1400 ms. All epochs were baseline-corrected with respect to the mean voltage over the 200 ms preceding the onset of stimulus, followed by averaging in association with experimental conditions irrespective of response (Liu et al., 2013).

This study focused on the ERPs elicited by disgusted, fearful, and neutral facial expressions in five duration conditions. We analyzed the amplitudes of fronto-central VPP and CNV, and occipito-temporal N170 components across different sets of electrodes according to grand-mean ERP topographies and relevant literatures (Monfort et al., 2000; Pouthas et al., 2000; Pfeuty et al., 2003a,b; N'Diaye et al., 2004; Gibbons and Rammsayer, 2005; Tarantino et al., 2010; Paul et al., 2011). The mean amplitudes of VPP were calculated at the electrode sites of FC1, FCz, and FC2 (time window = 170–190 ms). The mean amplitudes of N170 were calculated at P7, P8, PO7, and PO8 (time window = 170–190 ms). The CNV was analyzed using the data at FC1, FCz, and FC2, and were estimated using an area measurement which was calculated based on the integral under the ERP waveforms between two zero crossing points on the time axis (Macar et al., 1999; Macar and Vidal, 2003).

### Statistics

Statistical analysis was performed using SPSS Statistics 20.0 (IBM, Somers, USA). Descriptive data were presented as mean ± standard deviation (SD). The significance level was set at 0.05. Two-way repeated-measures ANOVAs were performed on the measurements of behaviors, the VPP amplitude, and the CNV area, with emotion (disgust, fear, and neutral) and stimulus duration (490, 595, 700, 805, and 910 ms) as within-subject factors. The three-way repeated measures ANOVA on the amplitudes of N170 component was conducted with emotion, stimulus duration, and hemisphere (left and right) as within-subject factors. Greenhouse-Geisser correction for ANOVA tests was used whenever appropriate. *Post-hoc* testing of significant main effects was conducted using Bonferroni method. Significant interactions were analyzed using simple effects model. Partial eta-squared (η^2^_p_) was reported to demonstrate the effect size in ANOVA tests, where 0.05 represents a small effect, 0.10 indicates a medium effect, and 0.20 represents a large effect.

## Results

### Behaviors

#### The proportion of “same” responses

**Figure 2:**
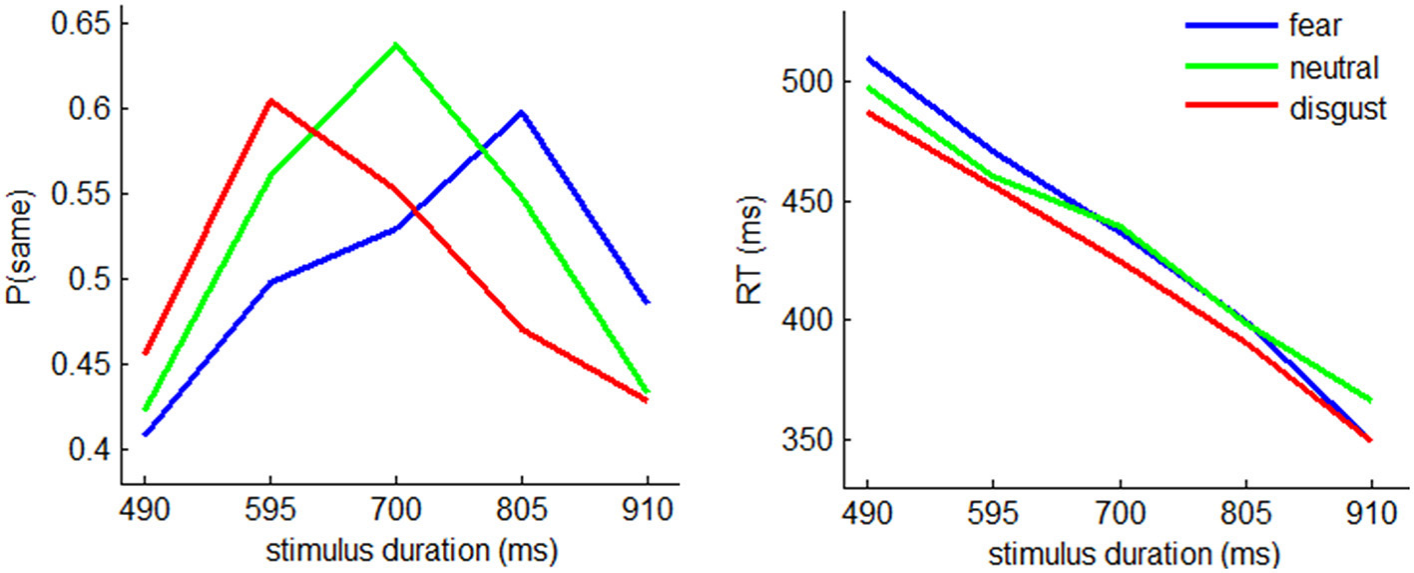
**The behavioral results. Left:** mean proportion of “same” responses in fear, neutral, and disgust conditions with the stimulus durations of 490, 595, 700, 805, and 910 ms. **Right:** mean reaction time.

In temporal generalization task, the accuracy is usually assessed by the proportion of “same” responses given by the participants (see also Pouthas et al., 2000; Gil and Droit-Volet, 2011). The interaction effect of emotion by stimulus duration was significant [*F*_(8, 224)_ = 4.07; *p* = 0.002; η^2^_p_ = 0.127]. Simple effect analysis showed that the proportion of “same” responses was modulated by emotion in 595- [*F*_(2, 56)_ = 4.02, *p* = 0.023], 700- [*F*_(2, 56)_ = 5.43, *p* = 0.007], and 805-ms conditions [*F*_(2, 56)_ = 6.36, *p* = 0.007] (Figure 2). In particular, 60% disgusted, 56% neutral, and 50% fearful faces with a duration of 595 ms were judged as with a duration of 700 ms while 47% disgusted, 55% neutral, and 60% fearful faces with a duration of 805 ms were judged as with a duration of 700 ms. This pattern of responses indicated that disgusted faces were estimated longer and fearful faces were estimated shorter when compared with neutral faces.

The main effect of stimulus duration was significant [*F*_(4, 112)_ = 12.8; *p* = 0.000; η^2^_p_ = 0.313]. The proportion of “same” responses was smaller in 490- (0.429 ± 0.208) and 910-ms conditions (0.449 ± 0.194), compared to 595- (0.554 ± 0.189), 700- (0.573 ± 0.182), and 805-ms conditions (0.539 ± 0.177) (*p*s < 0.021).

The main effect of emotion was not significant [*F*_(2, 56)_ < 1; *p* = 0.531; η^2^_p_ = 0.022].

#### Response time (RT)

The main effect of stimulus duration was significant [*F*_(4, 112)_ = 2.4; *p* = 0.000; η^2^_p_ = 0.889] (Figure 2). *Post-hoc* pairwise comparisons indicated that the RT measures in each pair of duration conditions showed significant differences (*p*s < 0.001). The RT decreased with increasing stimulus duration (533 ± 80.0 ms, 490 ± 79.1 ms, 465 ± 88.0 ms, 428 ± 84.4 ms, and 388 ± 76.4 ms in 490-, 595-, 700-, 805-, and 910-ms conditions).

The main effect of emotion was not significant [*F*_(2, 56)_ = 1.53; *p* = 0.225; η^2^_p_ = 0.052].

### ERPs

#### VPP

The interaction effect of emotion by stimulus duration was not significant [*F*_(8, 224)_ < 1; *p* = 0.676; η^2^_p_ = 0.025].

**Figure 3:**
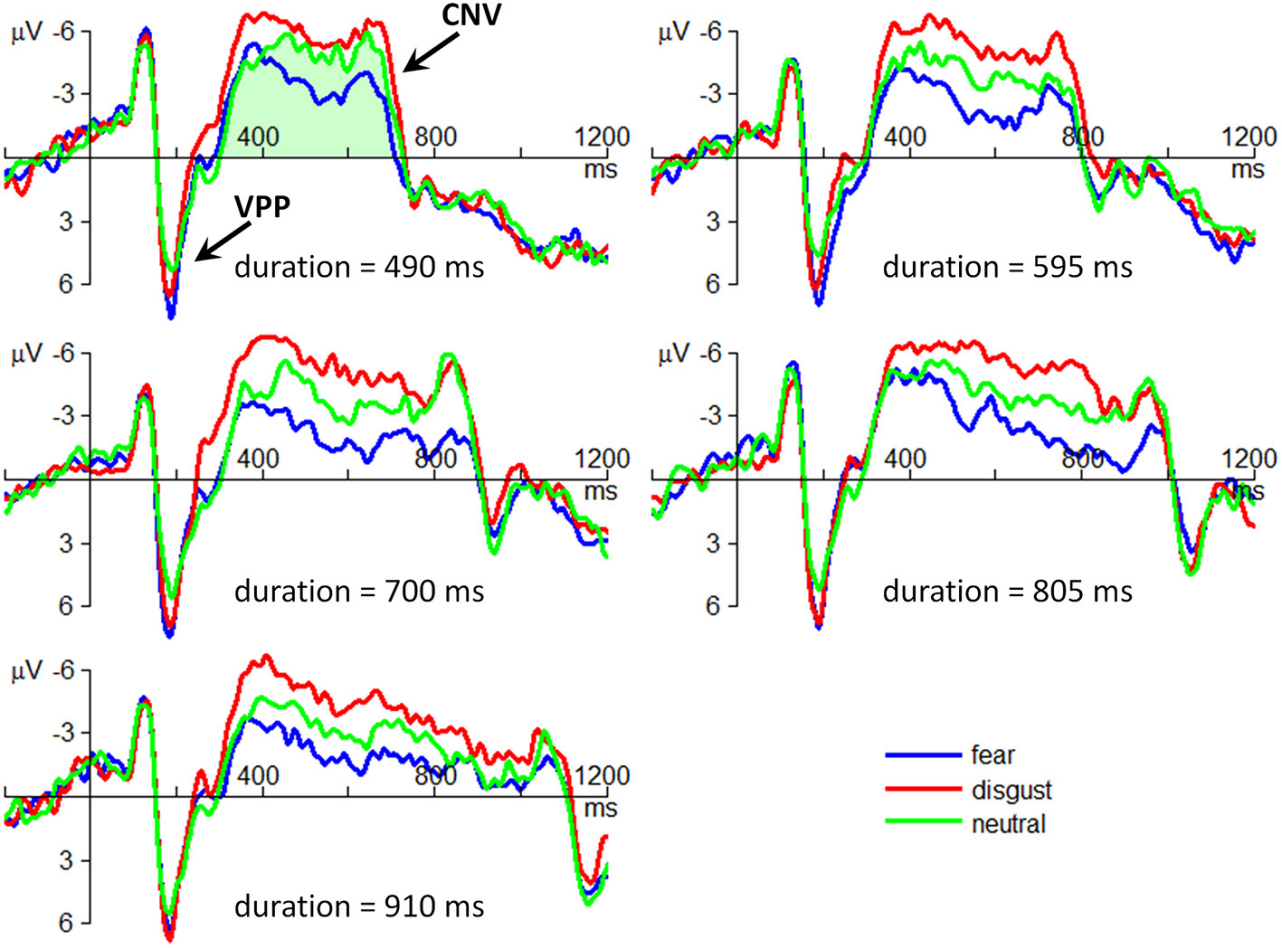
**The grand-mean ERP waveforms of the VPP and the CNV components**. All the plots are drawn using the data at the electrode site of FCz. The amplitudes of CNV were computed using area amplitude based on the integral under the ERP waveforms between two zero crossing points on the time axis. An example of CNV amplitude computation is shown as the light green region in the top left corner of the figure.

The main effect of emotion was significant [*F*_(2, 56)_ = 13.4; *p* = 0.000; η^2^_p_ = 0.325] (Figure 3). The VPP amplitude in neutral condition (4.75 ± 5.23 μV) was smaller than that in fear (6.76 ± 5.97; *p* = 0.001) and in disgust conditions (6.08 ± 5.42 μV; *p* = 0.002). No difference was found between fear and disgust conditions (*p* = 0.121).

The main effect of stimulus duration was not significant [*F*_(4, 112)_ = 1.50; *p* = 0.214; η^2^_p_ = 0.051].

#### N170

The interaction effect of emotion by hemisphere was significant [*F*_(2, 56)_ = 4.89; *p* = 0.011; η^2^_p_ = 0.149]. Simple effect analysis indicated that the effect of emotion on N170 was significant only at the right hemisphere [*F*_(2, 56)_ = 12.4; *p* < 0.001], with smaller N170 amplitudes in neutral condition (−5.09 ± 4.11 μV) compared with those in fear (−6.02 ± 4.52 μV; *p* < 0.001) and in disgust conditions (−5.91 ± 4.62 μV; *p* < 0.001). There was no significant effect of emotion at the left hemisphere [*F*_(2, 56)_ < 1].

**Figure 4:**
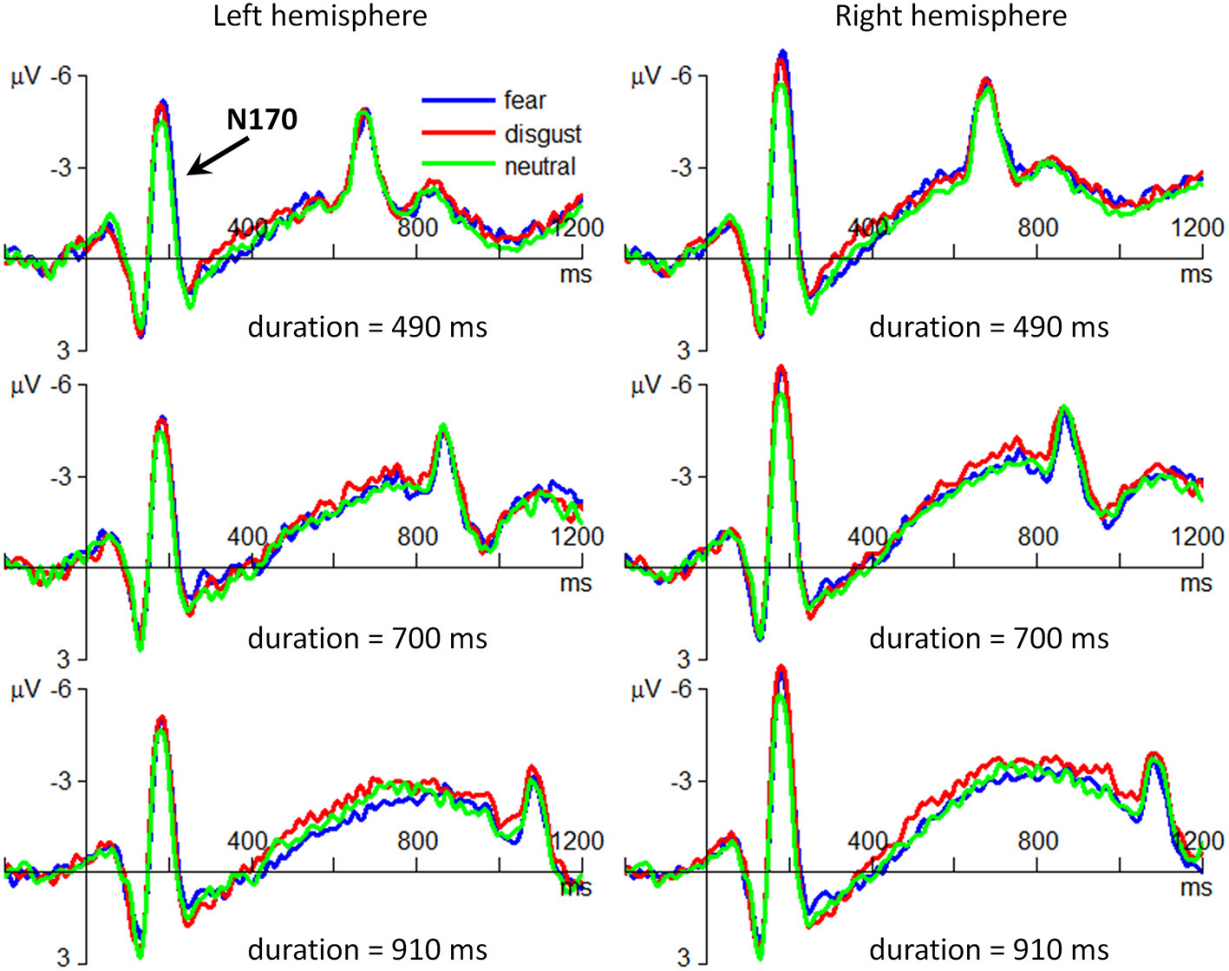
**The grand-mean ERP waveforms of the N170 component**. The three plots in the left column are drawn using the data at the electrode site of P7; the three plots in the right column are drawn using the data at P8. For the sake of brevity, only the ERPs in the three conditions of stimulus duration (490/700/910 ms) are displayed.

The main effect of emotion was significant [*F*_(2, 56)_ = 7.18; *p* = 0.002; η^2^_p_ = 0.204] (Figure 4). The N170 amplitude in neutral condition (−4.49 ± 4.41 μV) was smaller than that in fear (−5.00 ± 5.16; *p* = 0.007) and in disgust conditions (−4.91 ± 5.19 μV; *p* = 0.028). No difference was found between fear and disgust conditions (*p* = 1.000).

The main effect of stimulus duration was not significant [*F*_(4, 112)_ < 1; *p* = 0.822; η^2^_p_ = 0.013]. The main effect of hemisphere was not significant [*F*_(1, 28)_ = 2.46; *p* = 0.128; η^2^_p_ = 0.081].

#### CNV

**Figure 5:**
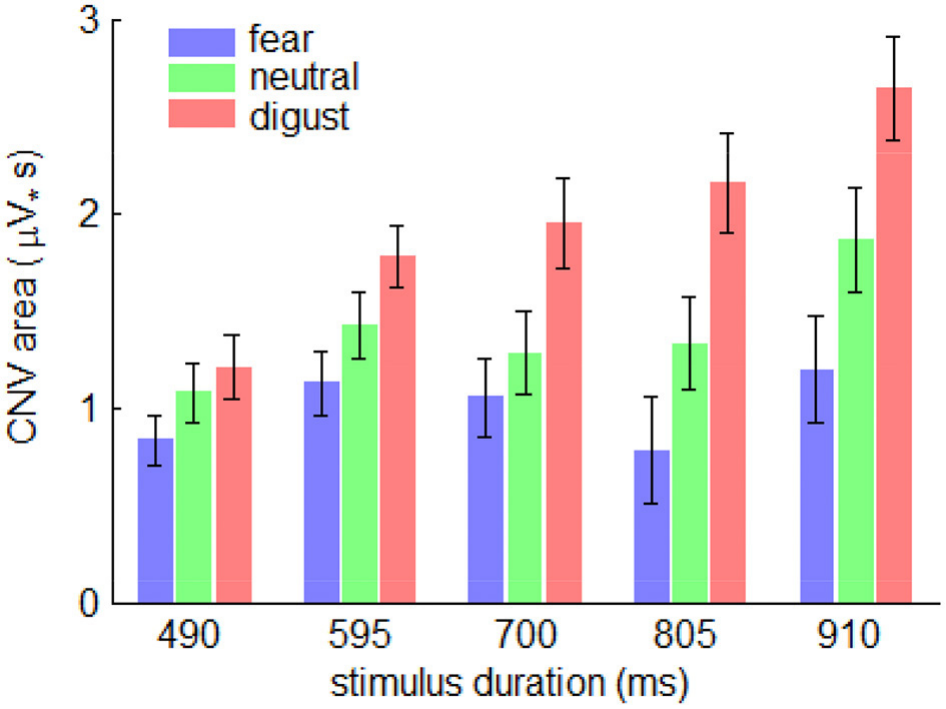
**The interaction effect of emotion by stimulus duration on CNV area**. Bars represent standard error of the mean.

The interaction effect of emotion by stimulus duration was significant [*F*_(8, 224)_ = 2.38; *p* = 0.037; η^2^_p_ = 0.078]. Simple effect analysis indicated that the effect of emotion on the CNV area first increased, then decreased with the increasing duration of stimulus presentation [*F*_(2, 56)_ = 3.76; *p* = 0.030; η^2^_p_ = 0.118 in 490-ms condition; *F*_(2, 56)_ = 4.72; *p* = 0.013; η^2^_p_ = 0.144 in 595-ms condition; *F*_(2, 56)_ = 7.74; *p* = 0.001; η^2^_p_ = 0.216 in 700-ms condition; *F*_(2, 56)_ = 10.7; *p* < 0.001; η^2^_p_ = 0.276 in 805-ms condition; *F*_(2, 56)_ = 9.60; *p* < 0.001; η^2^_p_ = 0.255 in 910-ms condition] (Figure 5).

The main effect of emotion was significant [*F*_(2, 56)_ = 21.9; *p* = 0.000; η^2^_p_ = 0.439] (Figure 3). The CNV area in fear condition (1.00 ± 2.26 μV_•_s) was smaller than that in neutral (1.40 ± 2.5 μV_•_s; *p* = 0.021) and in disgust conditions (1.94 ± 2.35 μV_•_s; *p* < 0.001). The CNV amplitude in neutral condition was smaller than that in disgust condition (*p* = 0.003).

The main effect of stimulus duration was significant [*F*_(4, 112)_ = 3.49; *p* = 0.032; η^2^_p_ = 0.111]. However, *post-hoc* pairwise comparisons showed no significant difference between duration conditions (1.10 ± 1.55, 1.44 ± 1.75, 1.38 ± 2.30, 1.42 ± 2.73, and 1.90 ± 2.30 μV_•_s in 490-, 595-, 700-, 805-, and 910-ms conditions).

## Discussion

The current study employed arousal-controlled facial expressions to examine the influence of threat-related emotions on time perception. Using neutral faces to provide a putative baseline, we demonstrated that disgust and fear have distinct influences on attention-related mechanism of time perception. At the behavioral level, the means of subjective timing depended greatly on the subtype of threat-related emotions, with disgusted faces being estimated longer and fearful faces being estimated shorter when both compared with neutral faces (Figure 2). Accordingly, the ERP data revealed that the CNV, which is an online index of timing, displayed separated waveforms in different emotional conditions, with larger amplitudes in disgust and smaller amplitudes in fear conditions when compared with neutral condition (Figure 3).

The behavioral finding of the underestimation of time in the fear condition was remarkable, because previous behavioral studies often found that the duration of emotional faces was overestimated compared with neutral faces (Droit-Volet et al., 2004; Effron et al., 2006; Gil et al., 2007; Mondillon et al., 2007; Tipples, 2008; Doi and Shinohara, 2009; Droit-Volet and Gil, 2009; Gil and Droit-Volet, 2011). In the pacemaker-accumulator model, inaccurate time perception is usually explained in terms of arousal-induced and attention-related mechanisms (Matell and Meck, 2000; Droit-Volet et al., 2004; Droit-Volet and Meck, 2007; Wittmann and Paulus, 2008). Within the arousal-induced mechanism, the physiological arousal level of emotional stimuli accelerates the pacemaker, leading to a greater number of accumulated pulses, thus resulting in overestimation of time (Angrilli et al., 1997; Droit-Volet et al., 2004; Droit-Volet and Meck, 2007; Gil et al., 2007; Noulhiane et al., 2007; Stetson et al., 2007; Tipples, 2008). After controlling the arousal ratings across the three emotional conditions, the present study mainly investigated the attention-related time distortions caused by fearful and disgusted faces. We hypothesized that, compared with neutral faces, fearful faces attract more attention to the emotional content of the stimuli and thus divert processing resources from the timer, resulting in an underestimation of time; in contrast, disgusted faces suppress the attention to the emotional content of the stimuli and thus could allocate more attentional resources to timing, resulting in an overestimation of time (Lerner and Keltner, 2000; Vuilleumier, 2005; Krusemark and Li, 2011). Consistent with this hypothesis, the behavioral data showed that the duration of fearful faces was judged shorter and the duration of disgusted faces was judged longer than that of neutral faces. Similarly, there were another two behavioral studies controlled the arousal levels of negative pictures (mainly fearful ones, e.g., spiders or rats) when investigating the effect of emotion on timing, which found that negative pictures were overestimated in high-arousal condition (arousal range = 6.5–7.5) while they were underestimated in low-arousal condition (arousal range = 4–5.7), compared with low-arousal neutral pictures (Angrilli et al., 1997; Gil and Droit-Volet, 2012). This opposite effect of negative pictures as a function of arousal suggested that the attention-related mechanism mainly works for low arousing stimuli whereas the arousal-induced mechanism mainly works for high arousing stimuli (Angrilli et al., 1997; Buhusi and Meck, 2006; Droit-Volet and Meck, 2007). We'd like to point out that this implication is in line with the design of the current study. It has been suggested that emotional facial expressions usually have smaller arousal compared with emotional pictures (Wangelin et al., 2012). Thus, we investigated in this study *per se* relatively low arousing stimuli of fear and disgust (arousal scores in this study: disgusted faces = 5.69 ± 0.45; fearful faces = 5.78 ± 0.52) so as to explore the attention-related mechanism of time perception.

This study also found that the early stage of emotion-modulated time perception was represented by larger fronto-central VPP amplitudes as well as larger occipito-temporal N170 amplitudes in fear/disgust conditions, compared with neutral condition. Furthermore, the effect of emotion of N170 were only significant in the right hemisphere, which is consistent with previous findings (Bentin et al., 1996; Luo et al., 2010; Zhang et al., 2012). Although many researchers have suggested that the VPP and the N170 are modulated by emotional faces, with larger amplitudes for emotional (e.g., fearful) than for neutral faces (Williams et al., 2006; Schyns et al., 2007; Zhang et al., 2013), most of the previous studies chose emotional faces with similar arousal but let them have higher arousal ratings than neutral faces (for examples, see Batty and Taylor, 2003; Williams et al., 2006; Blau et al., 2007). This manipulation reflects a natural co-variation between arousal and valence ratings of emotional stimuli (i.e., a U-shape relation in valence-arousal coordinate) (Lang et al., 1998). However, the emotion-modulation effects on ERP components found in these studies may result from the variations of either valence or arousal, or both. Thus, another contribution of the current study is to reveal that the valence effect of emotional facial expressions could independently enhance the amplitudes of the VPP and the N170.

The most novel finding of this study is that compared with neutral faces, disgusted faces evoked enlarged CNV area while fearful faces were followed by narrower CNV area. It has been proposed that the medial fronto-central cortex (typically at the electrode site of FCz), in particular the supplementary motor area (SMA), is the neural substrate of the timing function reflected by the CNV (Macar et al., 1999; Macar and Vidal, 2002, 2004; Pfeuty et al., 2005). The SMA shows consistent activation in temporal processing (Tanji, 1994; Macar et al., 1999; Coull et al., 2004, 2008). More importantly, increasing attention to timing selectively enhanced the activation of the SMA (Coull et al., 2004). In line with these arguments, it can be inferred that the level of neural activation contributing to time perception depends on the amount of attention paid to timing (Macar et al., 2002; Macar and Vidal, 2003, 2004). As a result, the observed larger CNV area in disgust condition reflected that less attention was paid to emotional processing so relatively more attentional resources were left for time processing. In contrast, smaller CNV area in fear condition reflected that more attention was paid to emotional processing so relatively less attentional resources were left for time processing. Therefore, despite both being threat-related, fear and disgust may engage opposite attention effects with contrasting CNV results. The major contribution of the current study is to provide neural correlates of such divergence in the aspect of time perception and to demonstrate beyond the behavioral level that the categorization of threat-related emotions should be refined so as to highlight the adaptability of the human defense system to optimize actions to diverse dangers in the environment (Krusemark and Li, 2011).

Finally, it should be noted that the interaction effect of emotion by stimulus duration on the CNV area displayed a non-linear pattern: the effect of emotion first increased, then decreased with the increasing duration of stimulus presentation, i.e., the effect of emotion on temporal perception seems to be larger for the intermediate durations (595, 700 and 805 ms) than for the extreme durations (490 and 910 ms). This CNV pattern is consistent with previous findings that the highest sensitivity for time perception locates in the range of 300 to 800 ms, especially at 600 ms or 700 ms (Drake and Botte, 1993; Zelaznik et al., 2000; McAuley et al., 2006). The CNV results obtained in this study implied that the attentional mechanism could not explain all of the variations of the data. Further studies are needed to examine the multiple factors that affect time perception.

## Conclusion

To sum up, the current study investigated and compared the effect of emotion of two subtypes of threat-related emotions on the judgment of time duration using arousal-controlled emotional stimuli. We demonstrated that disgust and fear had distinct influences on attention-related mechanism of time perception and thus resulted in different behavioral as well as ERP patterns. In particular, the perceived duration of disgusted faces was longer than that of neutral faces while fearful faces were estimated to be presented shorter than neutral faces. Accordingly, the CNV showed larger amplitudes in disgust and smaller amplitudes in fear conditions when compared with neutral condition. It is implicated that the categorization of threat-related emotions should be refined so as to highlight the adaptability of the human defense system to optimize actions to diverse dangers in the environment.

### Conflict of interest statement

The authors declare that the research was conducted in the absence of any commercial or financial relationships that could be construed as a potential conflict of interest.
